# Prognostic Significance of KIF11 and KIF14 Expression in Pancreatic Adenocarcinoma

**DOI:** 10.3390/cancers13123017

**Published:** 2021-06-16

**Authors:** Anna Klimaszewska-Wiśniewska, Izabela Neska-Długosz, Karolina Buchholz, Justyna Durślewicz, Dariusz Grzanka, Anna Kasperska, Paulina Antosik, Jan Zabrzyński, Alina Grzanka, Maciej Gagat

**Affiliations:** 1 Department of Clinical Pathomorphology, Faculty of Medicine, Collegium Medicum in Bydgoszcz, Nicolaus Copernicus University in Toruń, 85-094 Bydgoszcz, Poland; iznes@cm.umk.pl (I.N.-D.); karolina.buchholz@cm.umk.pl (K.B.); justyna.durslewicz@cm.umk.pl (J.D.); d_grzanka@cm.umk.pl (D.G.); anna.skorczewska@cm.umk.pl (A.K.); paulina.antosik@cm.umk.pl (P.A.); jan.zabrzynski@cm.umk.pl (J.Z.); 2 Department of Histology and Embryology, Faculty of Medicine, Collegium Medicum in Bydgoszcz, Nicolaus Copernicus University in Toruń, 85-092 Bydgoszcz, Poland; agrzanka@cm.umk.pl (A.G.); mgagat@cm.umk.pl (M.G.); 3 Department of General Orthopaedics, Musculoskeletal Oncology and Trauma Surgery, Poznan University of Medical Sciences, 60-572 Poznań, Poland

**Keywords:** pancreatic adenocarcinoma, prognostic factor, KIF11, KIF14, cell division, genomic instability

## Abstract

**Simple Summary:**

Prognostic markers for survival stratification of patients with pancreatic adenocarcinoma (PAC) are missing yet. Therefore, the primary aim of this study was to assess the expression, clinical associations, and survival implications of KIF11 and KIF14 in PACs. In addition, the genes co-expressed with KIF11 or KIF14 were predicted and functionally annotated. Herein, we found that the expression patterns of KIF11 and KIF14 alter significantly in PACs, at both protein and mRNA levels, and this may be harnessed for patient prognosis. KIF11 and KIF14 could be defined as positive prognostic biomarkers based on the protein-based immunohistochemistry data, while they were associated with adverse prognosis based on the transcriptomic data. We also captured a five-gene prognostic signature and the biology associated with it. The findings of the present study suggest that KIF11 or KIF14 proteins, as well as a new five-gene panel, may serve as potentially useful prognostic biomarkers for PAC.

**Abstract:**

Available biomarkers for pancreatic adenocarcinoma (PAC) are inadequate to guide individual patient prognosis or therapy. Therefore, herein we aimed to verify the hypothesis that differences in the expression of KIF11 and KIF14, i.e., molecular motor proteins being primarily implicated in cell division events could account for the differences in the clinical outcome of PAC patients. In-house immunohistochemistry was used to evaluate the protein expressions of KIF11 and KIF14 in PAC, whereas RNA-seq datasets providing transcript expression data were obtained from public sources. IHC and mRNA results were correlated with clinicopathological features and overall survival (OS). Furthermore, the genes co-expressed with *KIF11* or *KIF14* were predicted and functionally annotated. In our series, malignant ducts displayed more intense but less abundant KIF11 staining than normal-appearing ducts. The former was also true for KIF14, whereas the prevalence of positive staining was similar in tumor and normal adjacent tissues. Based on categorical immunoreactive scores, we found KIF11 and KIF14 to be frequently downregulated or upregulated in PAC cases, respectively, and those with elevated levels of either protein, or both together, were associated with better prognosis. Specifically, we provide the first evidence that KIF11 or KIF14 proteins can robustly discriminate between patients with better and worse OS, independently of other relevant clinical risk factors. In turn, mRNA levels of *KIF11* and *KIF14* were markedly elevated in tumor tissues compared to normal tissues, and this coincided with adverse prognosis, even after adjusting for multiple confounders. Tumors with low predicted *KIF11* or *KIF14* expression were seen to have enrichment for circadian clock, whereas those with high levels were enriched for the genomic instability-related gene set. *KIF11* and *KIF14* were strongly correlated with one another, and *CEP55*, *ASPM*, and *GAMT* were identified as the main hub genes. Importantly, the combined expression of these five genes emerged as the most powerful independent prognostic indicator associated with poor survival outcome compared to classical clinicopathological factors and any marker alone. In conclusion, our study identifies novel prognostic biomarkers for PAC, which await validation.

## 1. Introduction

The global burden of pancreatic cancer has been growing at an alarming pace over the past two decades, counting 458,918 new cases and causing 432,242 deaths (4.5% of all cancer deaths) in 2018. This ranks it only the twelfth most common cancer worldwide; however, given that the mortality rate almost equals the incidence rate, it accounts for the seventh leading cause of cancer-related deaths globally, the fourth in Europe, and the third in the USA. The future burden of pancreatic cancer is estimated to only increase and will include 355,317 new cases until 2040, surpassing breast, prostate, and colorectal cancers [1]. Pancreatic ductal adenocarcinoma (PDAC), the most common histology, accounts for 85–90% of these tumors. PDAC has an extremely poor prognosis, with the estimated median survival less than a year and overall 5-year survival rate less than 5% (range 2–9%), which is the lowest among all other malignancies. The reason behind such unfavorable prognosis is multifactorial and mostly related to its particularly aggressive biology, late-stage presentation, and a severe lack of effective therapeutics due to drug resistance [2].

In the context of unique pathobiology, several molecular events, including genetic alterations accompanied by deregulated signaling pathways, are now believed to be responsible for PDAC onset, progression, and malignant behavior. The great majority of PDACs are characterized by four genetic changes: the activation of *KRAS* and loss of *TP53, SMAD4*, and *CDKN2A.* Myriad additional genes are altered in subsets of tumors, but most of them still converge on few critical signal transduction pathways and cellular physiologies, of which cell cycle progression and cell motility have become eminently apparent, as it is the proliferative and invasive nature of malignant tumors that drives lethality [3].

The kinesin superfamily (KIF) comprises a group of closely related, microtubule (MT)-based and ATP-powered motor proteins, which are subdivided into 14 families, known to play an important role in many physiological processes, including cell division, motility and intracellular transport of vesicles, organelles, protein complexes, and mRNAs. KIFs are defined by their motor domains (heads) that contain both the MT and ATP binding sites and enable the conversion of the free energy of ATP into directed mechanical motion along microtubules. Based on the position of the motor domain, they are further subdivided into N-kinesin, C-kinesin, and M-kinesin groups [4]. Different members of the kinesin family, including KIF11 and KIF14, have been shown to be aberrantly expressed in a number of tumors, and also as a part of genomic instability (GIN) signatures [5]. Belonging to the kinesin-5 or kinesin-3 family, KIF11 and KIF14 are N-type and plus-end-directed motors with a primary role in the formation and maintenance of the bipolar spindle or cytokinesis, respectively. In addition, the functions of these kinesins have been shown to cover all phases of mitosis and most cell division-associated events [6]. It is, therefore, not surprising that the aberrant expression of KIF11 or KIF14 disturbs centrosome separation, bipolar spindle assembly, chromosome segregation, and cytokinesis, which eventually can result in GIN and the development and/or progression of a broad spectrum of cancers [7,8]. Both kinesins have been proposed to play non-mitotic functions as well, e.g., in axonal branching, growth cone motility, and cell migration. Consequently, these proteins are regarded as new promising targets for antimitotic drugs and as potential anti-invasive drugs, as well as prognostic biomarkers, for various groups of cancer patients [6,7,8]. However, their clinical utility in predicting survival outcomes in patients with PDAC is still unknown.

The present study was designed to investigate the expression of KIF11 and KIF14 in PDACs and adjacent tissues by semiquantitative immunohistochemistry (IHC) and to explore their correlation with clinicopathological features, as well as prognosis. We also applied transcriptomics of pancreatic adenocarcinoma patients and healthy donors using publicly available datasets to assess the expression levels and prognostic impact of KIF11 and KIF14 mRNAs. Functional enrichment analyses were further performed to predict biological functions and pathways related to KIF11 or KIF14, which may possibly impact the course of pancreatic adenocarcinoma.

## 2. Materials and Methods

### 2.1. Patients and Tissue Material

The cohort consisted of 68 patients with a histological diagnosis of pancreatic ductal adenocarcinoma who underwent a pancreatic resection at the Department of General, Hepatobiliary and Transplant Surgery, Collegium Medicum in Bydgoszcz, Nicolaus Copernicus University in Toruń (Poland), between 2009 and 2019. Variables of interest included gender, age, pathological T stage (pT), pathological N stage (pN), grading, staging, tumor location, vascular invasion (VI), perineural invasion (PNI), Ki-67 index, and MSH6 status. The primary source of Ki-67 values and MSH6 expression was our previous report [9]. The outcome of interest was overall survival (OS), which was defined as the time from diagnosis to last follow-up or death. Postsurgical survival data were available for 62 patients. The same cohorts of patients and tissue samples have been included in our previous article [9]; however, the final follow-up was extended to 30 April 2020. The median follow-up time was 1298 days, whereby 52 (83.87%) patients died during follow-up. PDACs were reclassified in accordance with the standardized TNM eighth edition classification of the American Joint Committee on Cancer (AJCC) criteria. Fifty-four of the 68 PDAC patients with the adjacent histologically normal pancreatic tissue, together with another 11 specimens from normal peritumoral tissue of other PDAC patients, were established to be the control group (*n* = 65). The use of tissues for this study was approved by the Institutional Ethics Committee of Nicolaus Copernicus University in Toruń, Collegium Medicum in Bydgoszcz (KB 342/2020).

### 2.2. Immunohistochemistry

Immunohistochemical staining was done on tissue macroarrays (TMA) constructed from a tumor-rich representative area of the paraffin blocks and the tumor-adjacent histologically normal tissue, as described previously [9]. Three- to four-micrometer-thin sections of the tissue arrays were dewaxed in xylene, rehydrated through graded alcohol concentrations, and antigen retrieved in either Ventana high-pH CC1 buffer (64 min for KIF11; Roche Diagnostics/Ventana Medical Systems, Tucson, AZ, USA) or Dako high-pH buffer (20 min for KIF14; Dako; Agilent Technologies, Inc., Santa Clara, CA, USA) in the automated PT Link system (Dako; Agilent Technologies). Slides were then transferred to the automated staining apparatus (either to the Dako Autostainer (DakoCytomation, Carpinteria, CA, USA) or BenchMark^®^ ULTRA (Roche Diagnostics/Ventana)) and endogenous peroxidase activity, as well as nonspecific binding sites, were quenched by 3% hydrogen peroxide and 3% bovine serum albumin (BSA), respectively.

Tissue arrays were incubated with a primary rabbit polyclonal anti-KIF11 antibody (1:150, 32 min; PA5-82394, ThermoFisher Scientific, Waltham, MA, USA) or primary rabbit polyclonal anti-KIF14 antibody (1:500, 30 min; HPA038061, Sigma Aldrich, St. Louis, MO, USA). Ki-67 and MSH6 IHC data generated on the same TMA were available from a previous study, and IHC was performed as previously published [9]. Primary antibodies were visualized using either the UltraView Universal DAB Detection Kit (Roche Diagnostics/Ventana) or the Envision Flex Kit (Dako, Agilent Technologies) followed by color development using 3,3-diaminobenzidine. Tissue sections were counterstained using hematoxylin, and finally cover-slipped in a Dako mounting medium (Agilent Technologies). Known positive control sections were applied for each antibody based on the antibody datasheet and the Human Protein Atlas (http://www.proteinatlas.org, accessed on 26 August 2020). As negative controls, the primary antibody was omitted, but all other steps were followed.

### 2.3. Immunohistochemical Scoring

All tumor images were reviewed using the light ECLIPSE E400 microscope (Nikon Instruments Europe, Amsterdam, the Netherlands), at 20× original objective magnification, by the experienced pathologist (I.N.D. or A.K.) with the help of a third one (D.G.) in some cases. All were blinded to the pathological and clinical data.

For a semiquantitative evaluation of the level of protein expression, proportion score, which reflected the fraction of positively stained cells (immunopercentage, PS), and intensity score, which represented the strength of staining (immunointensity, IS), were used. Immunointensity and immunopercentage were multiplied to yield a total expression score (immunoreactive score or immunoscore (IRS)), ranging from 0 to 12. The scores were recorded along the following criteria: score 0: no staining, score 1: weak positive, score 2: moderate positive, score 3: strong positive; score 0: <5% positive cells/areas, score 1: 5–24% positive cells/areas, score 2: 25–49% positive cells/areas, score 3: 50–74% positive cells/areas, and score 4: ≥75% positive cells/areas. In some cases, KIF11 and KIF14 expression was heterogeneous in PDAC tissues. Three fields (at × 20 magnification) were randomly chosen to evaluate staining, and mean IHC scores were calculated. All expression parameters (IS, PS, IRS) were dichotomized into “high” and “low” groups based on the optimal cut-point value defining with the cutp function of the Evaluate Cutpoints application [10], and these were correlated with clinicopathological features and outcomes (OS). High expression of KIF11 staining was defined by IRS ≥ 4.3, IS ≥ 2, and PS ≥ 2, whereas low KIF11 expression at the values of IRS < 4.3, IS < 2, and PS < 2. The cut-off values for KIF14 overexpression were defined at IRS ≥ 6, IS ≥ 1.5, and PS ≥ 4, while for KIF14 underexpression the cut-off points were established at IRS < 6, IS < 1.5, and PS < 4. To analyze the combined expression of KIF11 and KIF14, the KIF11 + KIF14 score was formed based on a total expression score (IRS). The KIF11 + KIF14 score was considered positive when expression of both KIF11 and KIF14 was high (KIF11^high^/KIF14^high^) and negative when both expression levels were low (KIF11^low^/KIF14^low^).

### 2.4. Database Analysis

The Cancer Genome Atlas (TCGA) cohort and Genotype-Tissue Expression (GTEx) cohort consisted of 177 samples diagnosed with pancreatic adenocarcinoma (146 of ductal histology) and 165 cases of non-cancerous pancreatic tissues, respectively. RNA-sequencing (RNA-seq) transcriptome data were downloaded through the UCSC Xena Browser (http://xena.ucsc.edu/ (accessed on 26 August 2020)) and normalized via DESeq2 normalization. mRNA expression was split into high-level or low-level groups according to the cut-off points determined with the Evaluate Cutpoints software [10]. Next, we investigated the top 50 genes that were positively or negatively correlated with *KIF11 or KIF14* in PAC using a web resource, UALCAN (http://ualcan.path.uab.edu/ (accessed on 19 April 2021)) [11] and the TCGA dataset. Pathway analysis and visualization were done using the Reactome pathway database (https://reactome.org (accessed on 19 April 2021)) [12], and Kyoto Encyclopedia of Genes and Genomes (KEGG) BRITE (https://www.genome.jp/kegg/brite.html (accessed on 19 April 2021)) was employed to capture functional hierarchies of *KIF11* or *KIF14* and the 50 top co-up- and co-downregulated genes.

### 2.5. Statistical Analysis

Statistical analysis was performed with the GraphPad Prism (version 7.01, GraphPad Software, La Jolla, CA, USA) and SPSS software packages (version 26.0, IBM Corporation, Chicago, IL, USA). Data normality was verified using the Shapiro–Wilk test. Categorical variables were compared using the chi-square (χ^2^) or Fisher’s exact test, and continuous variables were compared using the Mann–Whitney test. Spearman correlations were computed between the expression levels of individual markers. Overall survival curves were estimated using the Kaplan–Meier method, and differences in survival time were tested for significance using the log-rank test. Six patients who died post-operatively within 0–3 days of surgery were excluded from survival analyses. Univariate Cox proportional hazards analysis was performed to estimate the hazard ratio (and its 95% confidence interval (95% CI)) associated with each risk factor. Multivariate Cox proportional hazard analysis was done to control for confounder bias, and the hazard ratio (with 95% CI) was calculated. Adjustment variables included cell differentiation (well vs. moderate + poor), age at diagnosis (≤60 years vs. >60 years), gender (female vs. male), AJCC pathological stage (stage I + II vs. stage III + IV), pT (T1-T2 vs. T3-T4), pN (N0 vs. N1-N2), vascular invasion (absent vs. present), and perineural invasion (absent vs. present). Statistical tests were two-sided, with *p* < 0.05 considered significant.

## 3. Results

### 3.1. KIF11 and KIF14 Immunoexpression in Pancreatic Ductal Adenocarcinoma and Adjacent Normal Tissue

**Figure 1:**
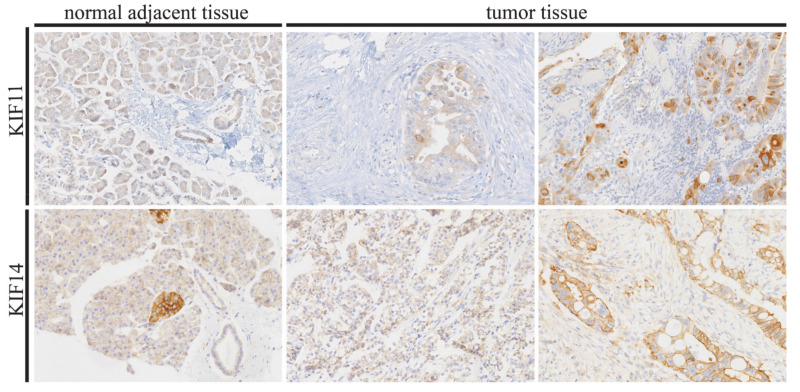
Representative images of immunohistochemical expression of KIF11 and KIF14 in pancreatic ductal adenocarcinoma and normal adjacent tissue (control). In tumor tissues, low expression (left images) and high expression (right images) of the studied proteins can be seen. Original magnification 20×.

KIF11 and KIF14 staining was localized to the cytoplasm and to the cytoplasm and/or membrane of tumor cells, respectively. In the case of KIF11, malignant ducts displayed generally more intense but less abundant staining than normal-appearing ducts. The former was also true for KIF14, whereas the prevalence of positive staining was similar in tumor and normal adjacent tissues. Representative photomicrographs of KIF11 and KIF14 immunoreactivity are shown in Figure 1.

**Figure 2:**
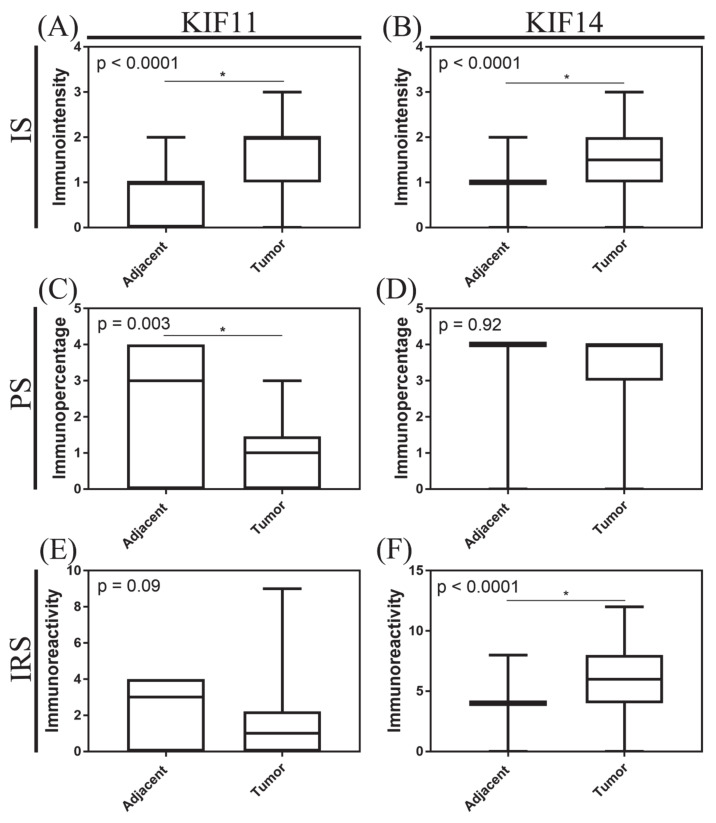
Immunoexpression of KIF11 and KIF14 in pancreatic ductal adenocarcinoma. The *X* axis of the plot represents histologically normal tissue that was adjacent to tumor tissue vs. cancer tissue, and the *Y* axis represents immunointensity, IS (**A**,**B**); immunopercentage, PS (**C**,**D**); or immunoreactivity, IRS (**E**,**F**). The top and bottom of the error bars represent the maximum and minimum values of data, respectively. The asterisk indicates statistical significance (* *p* < 0.05, Mann–Whitney test).

When analyzed as continuous variables, a significant increase in staining intensity was found for KIF11 and KIF14 in tumor tissue compared to normal adjacent tissue (both *p* < 0.0001, Mann–Whitney test; Figure 2A,B, respectively). In turn, the percentage of KIF11-stained cells was significantly lower in pancreatic ductal adenocarcinoma than in normal ducts (*p* = 0.003, Mann–Whitney test; Figure 2C), while for KIF14, there was no significant difference between both groups studied (*p* = 0.92, Mann–Whitney test; Figure 2D). The total expression scores for KIF11 tended to be decreased in tumor tissues relative to adjacent non-tumor tissues (p = 0.09, Mann–Whitney test; Figure 2E), whereas for KIF14, a statistically significant increase was found (*p* < 0.0001, Mann–Whitney test; Figure 2F). Descriptive statistics for KIF11 and KIF14 expression are presented in Table S1.

When the established definition of low and high expression for each category was applied, 42 (61.76%) and 47 (69.12%) patients demonstrated high-intensity staining tumors for KIF11 and KIF14, respectively. When the percentage of staining was scored, 10 (14.71%) and 37 (54.41%) patients had tumors that exhibited a high percentage of cells staining for KIF11 and KIF14, while the remaining 58 (85.29%) and 31 (45.59%) cases showed a low percentage of staining, respectively. Based on the formula for assessing the total immunoscore, 5 (7.35%) and 38 (55.88%) of patients had tumors that presented KIF11 and KIF14 overexpression; the remaining 63 (92.65%) and 30 (44.12%) cases displayed underexpression of these proteins, respectively.

### 3.2. Tumor Characteristics with Respect to KIF11 and KIF14 Immunoexpression

We next assessed whether KIF11 and KIF14 reactivity differed by clinicopathological features of PDAC patients. In the case of both proteins, neither the intensity score nor percentage score nor total immunoscore was significantly correlated with clinicopathological features (Table 1 and Table 2). However, the low staining extent of tumor cells for KIF11 was marginally significantly associated with the occurrence of perineural invasion (*p* = 0.05, Fisher’s exact test; Table 1).

### 3.3. Correlations between Protein Biomarkers

KIF11 and KIF14 immunoreactivity scores (Spearman r = 0.31; *p* = 0.01), but not percentage scores (Spearman r = 0.17; *p* = 0.16) were positively correlated with one another, while the correlation between intensity scores was of borderline significance (Spearman r = 0.24; *p* = 0.05). KIF11 expression via staining intensity (Spearman r = 0.28; *p* = 0.02) and the final immunoscore (Spearman r = 0.28; *p* = 0.02), but not percentage score (Spearman r = 0.21; *p* = 0.09), was significantly correlated with Ki-67 index. There were no significant correlations between either KIF14 IRS score (Spearman r = 0.17; *p* = 0.17) or PS score (Spearman r = - 0.07; *p* = 0.59) or IS score and Ki-67 index (Spearman r = 0.21; *p* = 0.09). All parameters of KIF11 and KIF14 expression, with the exception of KIF14-proportion score (Spearman r = 0.09; *p* = 0.47), were markedly related to MSH6 expression (*p* < 0.05). The complete results of Spearman correlation analysis can be seen in Figure S1.

### 3.4. Relationships to Survival

**Figure 3:**
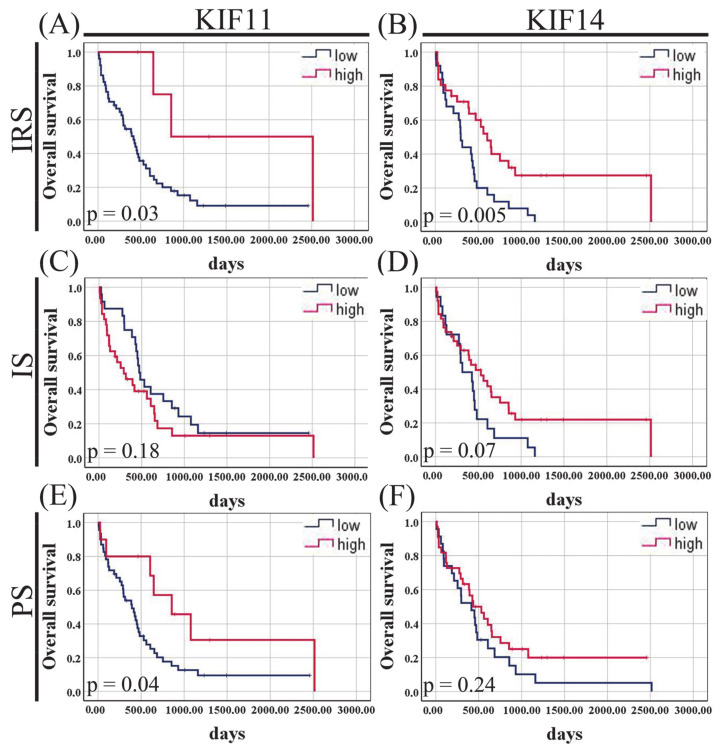
Kaplan–-Meier curves for overall survival of pancreatic ductal adenocarcinoma patients stratified by KIF11 (**A**,**C**,**E**) or KIF14 (**B**,**D**,**F**) immunoexpression. IS—immunointensity; PS—immunopercentage; IRS—immunoreactive score. *p*-Values were calculated using the log-rank test.

To determine whether levels of KIF11 or KIF14 protein expression might be associated with disease outcome in PDAC patients, we examined IRS, IS, and PS scores relative to OS in our in-house cohort. The Kaplan–Meier curves indicated that there was a significant difference in OS between PDAC patients stratified by KIF11 immunoscore (415 vs. 856, *p* = 0.03, log-rank test; Figure 3A) and percentage score (393 vs. 856, *p* = 0.04, log-rank test; Figure 3E), with better OS for high-expression groups. Notably, there was a trend for worse clinical outcomes for PDAC patients with increased staining intensity for KIF11; however, this was not a significant survival difference (290 vs. 471, *p* = 0.18, log-rank test; Figure 3C). KIF14 expression via IRS score was significantly related to the duration of OS, with better survival for the high-expression group (606 vs. 297, *p* = 0.005, log-rank test; Figure 3B). A similar tendency was found for KIF14 staining intensity, but without reaching statistical significance (531 vs. 314, *p* = 0.07, log-rank test; Figure 3D). KIF14 immunopercentage failed to stratify PDAC patients into significantly different survival groups (436 vs. 415, *p* = 0.24, log-rank test; Figure 3F). Of note, the best OS could be seen for patients whose PDACs were positive for KIF11 + KIF14 score, whereas patients with a negative score had significantly decreased OS (2515 days vs. 294 days, *p* = 0.004; Figure S2A). In addition, the association between KIF11 expression and overall survival was also explored when intensity and proportion scores were treated as continuous variables, and the results are presented in Figure S2B,C.

In the univariate Cox regression analysis (Table 3), the factors related to the duration of OS were high KIF11 IRS score (HR = 0.23, 95% CI 0.06–0.96, *p* = 0.04), high KIF11 PS score (HR = 0.41, 95% CI 0.17–0.98, *p* = 0.045), high KIF14 IRS score (HR = 0.44, 95% CI 0.24–0.79, *p* = 0.006), advanced tumor stage (HR = 2.05, 95% CI 1.02–4.13, *p* = 0.04), and vascular invasion (HR = 2.41, 95% CI 1.17–4.98, *p* = 0.02). When examined in the multivariate Cox analysis, KIF11 IRS (adjusted HR = 0.06, 95% CI 0.005–0.64, *p* = 0.02) and KIF14 IRS (adjusted HR = 0.20, 95% CI 0.08–0.51, *p* = 0.001) scores persisted as independent prognostic factors for OS (Table 4). Vascular invasion remained the only significant predictor of the established clinical parameters for OS of PDAC patients after adjusting for IRS or IS expression parameters and clinicopathological factors (Table 4). Notably, after correction for the bias caused by the univariate analysis, elevated KIF11 (HR = 3.05, 95% CI 1.29–7.19, *p* = 0.01) and KIF14 (HR = 0.25, 95% CI 0.10–0.67, *p* = 0.006) immunointensities were able to predict the clinical endpoint (OS) independently of age at diagnosis, gender, tumor grade, clinical stage, pN, pT, PNI, and VI.

### 3.5. KIF11 and KIF14 mRNA Expression in Pancreatic Adenocarcinoma and Normal Tissue Based on TCGA Datasets

**Figure 4:**
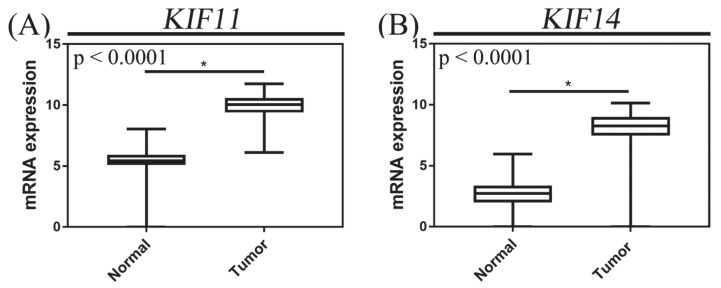
mRNA expression of KIF11 (**A**) and KIF14 (**B**) in pancreatic adenocarcinoma. mRNA expression data were retrieved from the Cancer Genome Atlas (TCGA); the *X* axis of the plot represents normal vs. cancer tissue, and the *Y* axis represents normalized expression of mRNAs. The top and bottom of the error bars represent the maximum and minimum values of data, respectively. The asterisk indicates statistical significance (* p < 0.05, Mann–Whitney test).

We next examined publicly available expression data on normal pancreatic tissue and pancreatic adenocarcinoma from GTEx and TCGA, respectively. We found that the expression of KIF11 and KIF14 mRNA in PACs was significantly upregulated compared with normal pancreatic tissues (both *p* < 0.0001, Mann–Whitney test; Figure 4), and high expression levels were demonstrated in 91 (51.41%) and 49 (27.68%) cases, respectively.

### 3.6. Correlations between Variables

In the TCGA cohort of PAC patients, the prevalence of KIF11 and KIF14 overexpression increased significantly with increasing histological grade (p = 0.01 and p = 0.002, respectively, χ^2^ test; Table 5*)*. KIF11 and KIF14 mRNA levels were significantly correlated with each other (Spearman r = 0.85; *p* < 0.0001), aneuploidy score (Spearman r = 0.34; *p* < 0.0001 and r = 0.37; *p* < 0.0001, respectively), fraction of genome altered (Spearman r = 0.42; *p* < 0.0001 and r = 0.41; *p* < 0.0001, respectively), and MKI67 expression (Spearman r = 0.95; *p* < 0.0001 and r = 0.83; *p* < 0.0001, respectively).

### 3.7. Functional Enrichment Analysis

**Figure 5:**
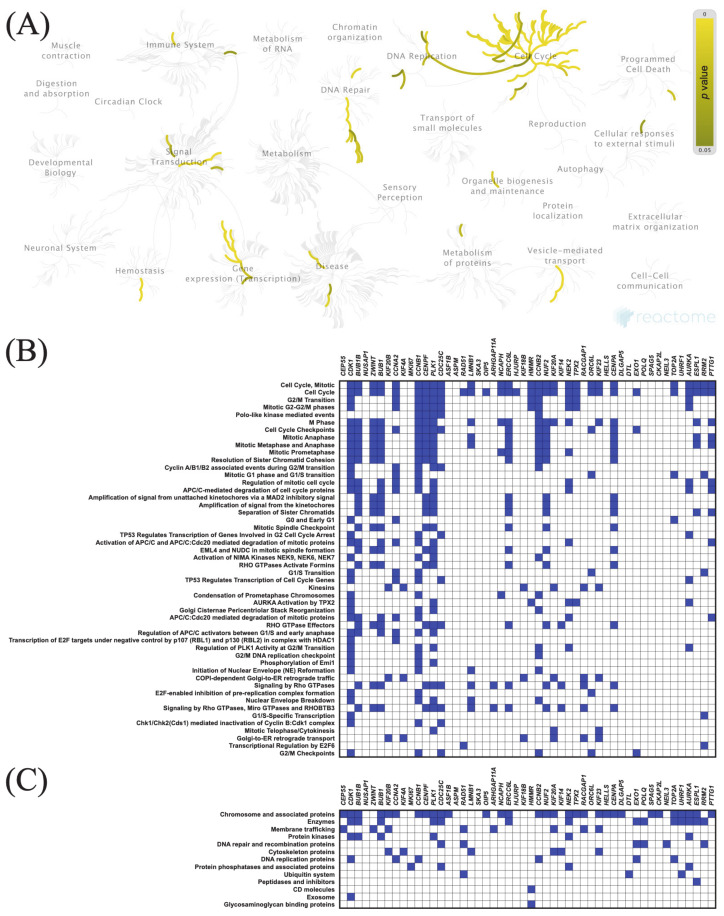
Functional enrichment analysis based on the TCGA dataset and UALCAN web tool. (**A**,**B**) The top 50 genes and Reactome pathways positively correlated with *KIF11* expression; (**C**) BRITE functional hierarchies for the top 50 genes that were co-upregulated with *KIF11*.

**Figure 6:**
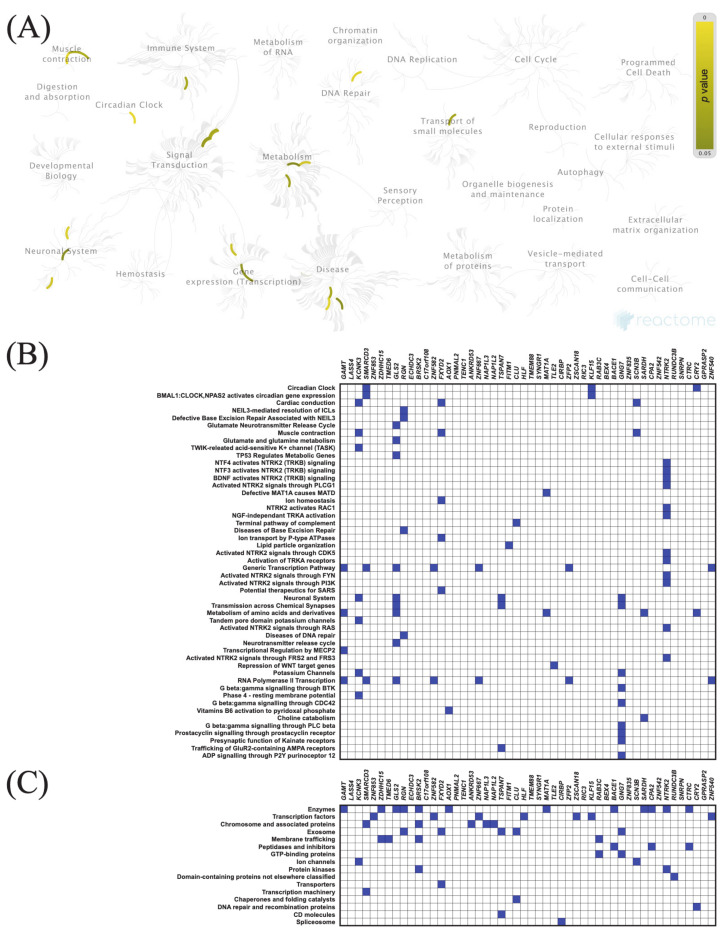
Functional enrichment analysis based on the TCGA dataset and UALCAN web tool. (**A**,**B**) The top 50 genes and Reactome pathways negatively correlated with *KIF11* expression; (**C**) BRITE functional hierarchies for the top 50 genes that were co-downregulated with *KIF11*.

**Figure 7:**
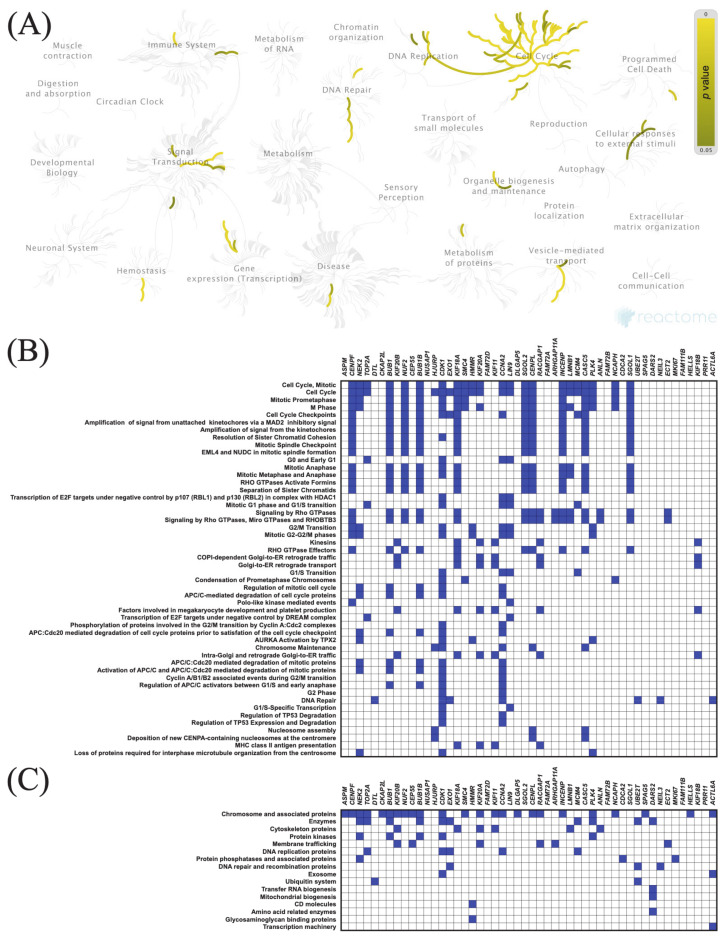
Functional enrichment analysis based on the TCGA dataset and UALCAN web tool. (**A**,**B**) The top 50 genes and Reactome pathways positively correlated with *KIF14* expression; (**C**) BRITE functional hierarchies for the top 50 genes that were co-upregulated with *KIF14*.

**Figure 8:**
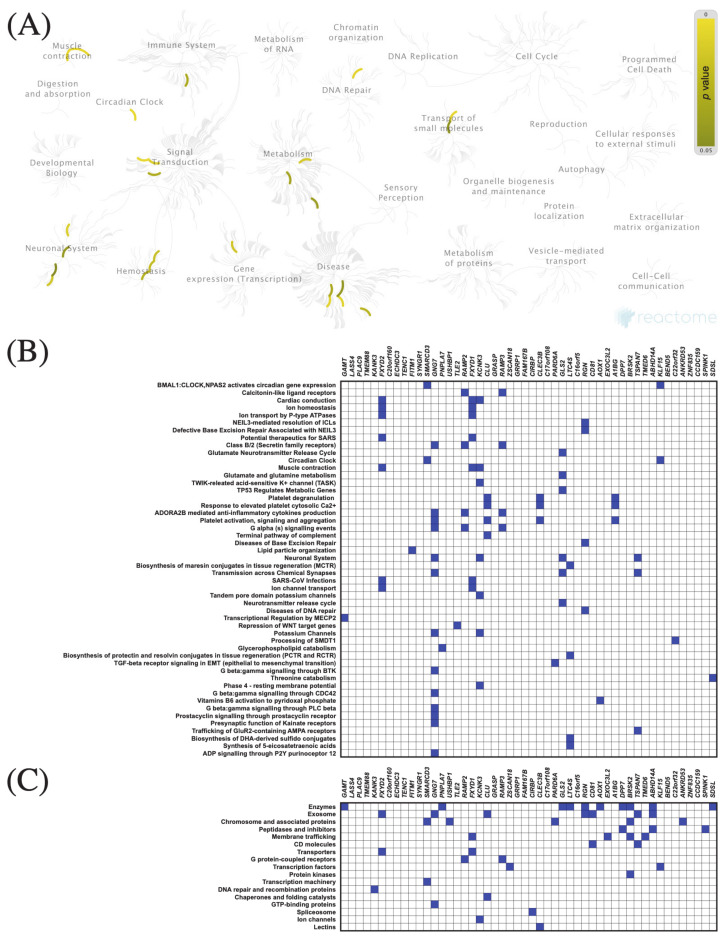
Functional enrichment analysis based on the TCGA dataset and UALCAN web tool. (**A**,**B**) The top 50 genes and Reactome pathways negatively correlated with *KIF14* expression; (**C**) BRITE functional hierarchies for the top 50 genes that were co-downregulated with *KIF14*.

The top 50 genes that were positively or negatively correlated with *KIF11* or *KIF14* in PAC samples were identified using the TCGA dataset and UALCAN web tool. Centrosomal Protein 55 (*CEP55*) and abnormal spindle-like microcephaly-associated protein (*ASPM*) were the top positively correlated genes with *KIF11* and *KIF14*, respectively, whereas guanidinoacetate N-methyltransferase (*GAMT*) was the top negatively correlated one (*KIF11:* Figure 5B and Figure 6B; *KIF14*: Figure 7B and Figure 8B). To confirm these correlations, we also examined the TCGA data of PAC patients via the UCSC Xena database, and the results showed positive correlations between *KIF11* and *CEP55* (r = 0.94; *p* < 0.0001), as well as between *KIF14* and *ASPM* (r = 0.96; *p* < 0.0001). In turn, *GAMT* was confirmed to be negatively correlated with *KIF11* (r = −0.42; *p* < 0.0001) and *KIF14* (r = −0.42; *p* < 0.0001). We next performed Reactome and KEGG BRITE enrichment analyses to determine the putative functions of *KIF11* or *KIF14* and the co-deregulated genes in PAC pathology. Reactome pathway analysis for *KIF11* showed that the co-upregulated genes were mainly involved in cell cycle, mitotic events, and G2/M transition (Figure 5A,B), while the downregulated genes were enriched in circadian clock (Figure 6A,B). For *KIF14*, the co-upregulated genes were primarily associated with cell cycle, mitotic events, and mitotic prometaphase (Figure 7A,B), while the co-downregulated genes were enriched in “BMAL1:CLOCK,NPAS2” and “calcitonin-like ligand receptors” (Figure 8A,B). KEGG BRITE functional hierarchies for *KIF11* revealed that among the co-upregulated genes, there were mostly “chromosome and associated proteins,” “enzymes,” “membrane trafficking proteins,” “protein kinases,” “DNA repair and recombination proteins,” “cytoskeleton proteins,” and “DNA replication proteins” (Figure 5C), whereas the co-downregulated genes had a preponderance of genes representing “enzymes,” “transcription factors, “exosome proteins,” “membrane trafficking proteins,” “peptidases and inhibitors,” “GTP-binding proteins” (Figure 6C). Similar results were obtained for *KIF14*, and the details are shown in Figure 7C and Figure 8C.

### 3.8. Relationships to Survival

**Figure 9:**
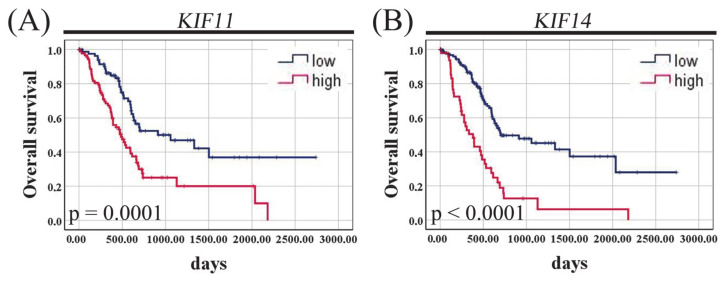
***Figure 9.*** Kaplan–Meier curves for overall survival of pancreatic adenocarcinoma patients of the TCGA cohort stratified by *KIF11* (**A**) or *KIF14* (**B**) mRNA expression. *p*-Values were calculated using the log-rank test.

The Kaplan–Meier curves demonstrated that PAC patients with high *KIF11* mRNA expression had shorter OS than those with *KIF11* underexpression (485 days vs. 913 days; *p* = 0.0001, log-rank test; Figure 9A). Likewise, high *KIF14* mRNA expression was significantly associated with poor OS (381 days vs. 702 days; *p* < 0.0001, log-rank test; Figure 9B).

Furthermore, patients whose PAC co-expressed *KIF11* and *KIF14* at high level had significantly shorter survival time compared to those patients whose tumor tissue simultaneously expressed both these markers at low level (1059 days vs. 334 days; *p* < 0.0001, log-rank test; Figure S3A), and the combined expression of two markers was only slightly more valuable for predicting prognosis than each marker individually (univariate Cox analysis: HR = 3.41, 95% CI 2.12–5.47; *p* < 0.0001; Table S2).

The univariate Cox analysis identified *KIF11* (HR = 2.25, 95% CI 1.47–3.45; *p* = 0.0002), *KIF14* (HR = 2.99, 95% CI 1.97–4.54; *p* < 0.0001), histological grade (HR = 2.18, 95% CI 1.15–4.13; *p* = 0.02), pN (HR = 2.10, 95% CI 1.25–3.52; *p* = 0.005), and pT (HR = 2.21, 95% CI 1.14–4.28; *p* = 0.02) as significant prognostic variables for OS (Table 5). The multivariate Cox analysis confirmed *KIF11* (adjusted HR = 1.75, 95% CI 1.13–2.70; *p* = 0.01), *KIF14* (adjusted HR = 2.65, 95% CI 1.70–4.15; *p* < 0.0001), and pN (adjusted HR = 2.00, 95% CI 1.15–3.47; *p* = 0.01) as independent prognostic factors for predicting OS (Table 6).

Since *CEP55*, *ASPM*, and *GAMT* were found to be hub genes in overexpressed and underexpressed networks, we also decided to verify the prognostic significance of this newly established 5-gene signature. The Kaplan–Meier analysis demonstrated that patients whose PACs simultaneously expressed *KIF11*, *KIF14*, *CEP55*, and *ASPM* at low levels and *GAMT* at high level had the best OS, and median OS time was not reached for this combination (unreached vs. 334 days; *p* < 0.0001, log-rank test; Figure S3B). Furthermore, the expression profile of *KIF11*^high^/*KIF14*^high^/*CEP55*^high^/*ASPM*^high^/*GAMT*^low^ had the highest prognostic hazard ratio values compared to each marker as a single indicator, as demonstrated in the univariate (HR = 5.18, 95% CI 2.63–10.20; *p* < 0.0001) and multivariate (HR = 5.21, 95% CI 2.41–11.27; *p* < 0.0001) Cox analyses of the TCGA PAC dataset (Table S2).

## 4. Discussion

In the present study, we aimed to determine the utility of KIF11 and KIF14 as markers of pancreatic cancer prognosis by analyzing the in-house IHC data and RNA-seq-based publicly available datasets in relation to clinicopathological traits and overall survival. Herein, we have found that the expression patterns of KIF11 and KIF14 alter significantly in PDAC, at both protein and mRNA levels, and this may be harnessed for patient prognosis.

Previous reports on pancreatic cancer models have demonstrated that all of the studied pancreatic cancer cell lines exhibited KIF11 overexpression [13,14,15], and that the oncogenic effect of this mitotic kinesin was associated with the promotion of cancer cell proliferation, colony formation, and tumor formation in mice [14,15]. In this context, Liu et al. have proposed a model in which KIF11 expression might be implicated in the pathogenesis of pancreatic cancer through the induction of GIN [15]. Furthermore, other studies of this group have revealed that a specific KIF11 inhibitor effectively suppressed cell proliferation and induced apoptosis in pancreatic cancer cell lines and tumor xenografts [14], as well as pancreatic cancer cell migration and invasion in vitro [13]. Apart from this experimental evidence, Liu et al. have further shown by IHC that KIF11 was overexpressed in PAC clinical specimens (*n* = 95) compared to normal pancreatic tissue samples (*n* = 10), tissues adjacent to pancreatic adenocarcinomas (*n* = 10), and benign pancreatic cystadenomas (*n* = 12) [15]. Moreover, in bioinformatics [16] and large-scale proteomic studies [17], KIF11 has been identified as one of the differentially expressed genes or proteins between PDAC and normal pancreas. The above-cited studies highlight a value of KIF11 as candidate drug target in pancreatic cancer, which awaits further evidence from clinical samples. However, less is known about KIF11 associations with clinicopathological parameters of PDACs [15], and even less about its effect on patient survival. To our knowledge, the present study is the first to evaluate the latter issue in PDAC. Based on our IHC results, it seems that the clinical relevance of KIF11 expression in PDACs may be more complicated than these results from in vitro and in vivo experiments. Indeed, we observed that malignant ducts displayed generally more intense but less abundant KIF11 staining than normal-appearing ducts and that both frequency and intensity differentially modulated survival outcome. Specifically, high KIF11 immunointensity was a frequent event in PDAC (*n* = 42/68; 61.76%), and it emerged as an independent predictor of poor OS after correction for the established prognostic factors. The vast majority of PDAC tissues (*n* = 58; 85.29%) were characterized by a low percentage of KIF11-positive cells, and on univariate analyses, this was significantly associated with a worse OS. When an integrated IRS score was applied, most tumors had low KIF11 expression (92.65%, *n* = 63), and these were associated with an increased risk for death, independent of multiple confounders. Furthermore, there was no association of KIF11 IRS score with any of the clinicopathological features, further suggesting its independence from potential confounders. Only 7.35% of the cases had KIF11 overexpression, whereby this expression pattern included patients whose tumors simultaneously exhibited high KIF11 staining intensity (mostly strong 3+ staining) on at least 25–49% of tumor cells. This coincided with superior prognosis, both in univariate and multivariate analysis. This raises the question of whether or not a high intensity of KIF11 staining is definitely associated with poor prognosis in PDAC. Analysis of the continuous KIF11 IS score with respect to OS seems to partially answer this question. In our series, the best median OS was observed for patients whose tumors exhibited weak staining intensity (854 days), albeit individuals with strong positive staining (647 days) had also a relatively better OS than those with moderate positive staining, who presented dramatically decreased duration of survival (254 days). Accordingly, increasing the threshold for determining high KIF11 immunointensity demonstrated a non-significant trend towards a favorable OS (647 days vs. 426 days, *p* = 0.25; Figure S2D). Based on these considerations, we conclude that KIF11 staining intensity provides imperfect prognostic information in PDAC, while the scoring system combining percent positivity and staining intensity is the most predictive for prognosis, and it is high KIF11 expression that acts as an independent prognostic indicator of improved OS. The latter finding contradicts the reported poor prognosis of breast [18], hepatocellular [19], clear cell renal cell [20], lung [16], and ovarian [21] cancers overexpressing KIF11. Limitations must be noted, as the robustness of our survival analysis might have been impaired by a limited number of cases in the high-expression group and incomplete patient data. Although our case number is within the limit adopted in other studies [22], 10 events per variable (EPV) has been previously recommended for a more powerful proportional hazards regression analysis [23]. Therefore, our findings need to be validated with a larger sample size. Furthermore, although the present study shows the association of KIF11 expression with PDAC outcome, this does not imply that a direct causal relationship exists. Specifically, a mechanistic explanation for the clinical significance of the predominant expression pattern of KIF11 in PDAC, i.e., moderate intensity (median IS = 2) on a low percentage of tumor cells (median PS = 1), remains elusive. Also, the finding that KIF11 OS association may vary between moderately and strongly stained PDACs needs to be empirically explained. However, given that a total immunoreactive score allowed us to categorize most PDACs as KIF11-downregulated tumors, and these had an adverse prognosis, it is a functional role of KIF11 in controlling genome integrity [15,24] that would potentially explain some of the pro- and anti-tumorigenic effects of this protein depending on the context and expression strength. In agreement with this, our cohort and TCGA studies demonstrated that KIF11 expression in pancreatic tumor tissues was indeed positively correlated with GIN markers, including MSH6, aneuploidy score, and fraction of genome altered. Further supports came from our gene set enrichment analyses, in which PACs with low predicted *KIF11* expression were observed to have enrichment for circadian clock, whereas those with its high levels were enriched for the genomic instability gene set, such as the genes involved in cell cycle, chromosome segregation, checkpoints, and DNA replication and repair. Recent studies have revealed that a tight crosstalk exists between genome maintenance pathways and circadian rhythm, and the alterations in circadian clock perturb genome integrity by modulating the cell cycle timing, altering DNA replication fork progression, influencing DNA damage response, and DNA repair efficiency [25]. However, as with any high-throughput studies, in silico data should be used as a hypothesis-generating tool, which still requires wet lab-based confirmation and mechanistic follow-up. Thus, there must be more research to validate these networks in the pathology of pancreatic adenocarcinoma.

KIF14 seems another cancer biomarker that fits well the features of a double-faced protein depending on, e.g., tumor type, cell context, expression levels, and interacting networks, as results across studies are heterogeneous. Based on dichotomized IRS scores, our cohort study revealed that KIF14 protein might be more frequently upregulated (55.88%) than downregulated (44.12%) in pancreatic ductal adenocarcinoma tissues, and that the former expression status independently conferred a better prognosis. These findings contradict the reported poor prognosis of medulloblastoma [26], cervical [27], gastric [28], hepatocellular [29], ovarian [30], and prostate [31] cancers overexpressing KIF14, but are in line with the correlation of high levels of KIF14 with a favorable prognosis of lung cancer [32]. They are also in partial agreement with the study by Abiatari et al. [33], who have shown the anti-invasive function of KIF14 in pancreatic cancer. In more detail, KIF14 was found to be strongly expressed in PDAC cells that did not invade nerves, whereas it was downregulated in those that did, as well as in non-invasive vs. neuroinvasive pancreatic carcinoma cell lines. Furthermore, knockdown of KIF14 increased invasiveness and resistance to anoikis of T3M4 pancreatic cancer cells [33]. Although we failed to find the association of KIF14 positivity with PNI in our cohort, it is still possible that overexpression of KIF14 protein in PDAC tissues could be a negative feedback mechanism counteracting tumor invasiveness [33].

Through data analysis with the Cancer Genome Atlas (TCGA), we then identified elevated *KIF11* and *KIF14* mRNA levels of pancreatic adenocarcinomas as independent predictors of reduced survival. These results correspond to the in silico gene expression analysis by Wu et al., who revealed that *KIF14*, among other genes, was upregulated and positively associated with a poor survival of PDAC patients [34]. Furthermore, *KIF14* expression was found to be elevated in PDAC cDNA microarray datasets from the Oncomine, which were analyzed in the study by Suh et al. [35]. In turn, for KIF11, Liu et al. found that its mRNA levels were significantly elevated in PDAC tissues with gene copy number gain being a potentially important contributor [15]. In the TCGA cohort, we further demonstrated that not only were *KIF11* and *KIF14* overexpressed in PAC tissues in a grade-dependent fashion, but they were also strongly correlated with MKI67 expression. These observations are in agreement with previous non-pancreas data [36,37,38]. Thus, our study indicated that KIF11 and KIF14 could be defined as good prognostic markers based on the protein-based IHC data, while they were associated with adverse prognosis based on the transcriptome data. Correlations between mRNA and protein data have been extensively investigated and debated in recent years, and it is clear that in many situations measurements of mRNA levels alone cannot be relied upon to provide an accurate reflection of protein abundance [39]. Specifically, it has been suggested that regulators of cellular division and differentiation would be expected to be enriched for negative correlations between mRNA and protein levels. Systematic research has further displayed multiple processes beyond the “non-correlation” between protein and mRNA expression patterns, which may result in the opposite prognostic significance [40,41]. A possible explanation for the discrepancy in the prognostic value of mRNA and protein expression of KIFs, found in our research, is the influence of post-transcriptional, translational, and degradation regulation on protein abundance. This study design, however, is not intended to investigate the association between mRNA and protein levels of KIF11 and KIF14 because they were evaluated in two different patient cohorts, which is certainly another limitation of our research. Based on the present study, we may, however, conclude that the difference in prognostic value of protein versus transcript may be explained, at least in the case of KIF11, by the observed discordance between protein and mRNA expression levels. Thus, the underlying mechanisms that regulate mRNA transcription of *KIF11* and/or are responsible for poor protein/mRNA abundance correlation in PDACs are putative offenders leading to unfavorable survival outcome.

We next defined *CEP55*, *ASPM*, and *GAMT* as the main hubs, which were closely related to *KIF11* and *KIF14* in PAC. Furthermore, *KIF11* and *KIF14* mRNA levels were strongly correlated with one another in pancreatic tumor samples from the TCGA cohort, which could be also observed in our functional enrichment. The correlation between KIFs was also validated at the protein level in our cohort. Consequently, we assumed that this putative biological connection in PAC could be reflected in an increase of prognostic power of the newly established 5-gene signature. Indeed, we found that a combined expression profile of *KIF11*^high^/*KIF14*^high^/*CEP55*^high^/*ASPM*^high^/*GAMT^l^*^ow^ emerged as the most powerful independent prognostic indicator associated with poor survival outcome compared to classical clinicopathological factors, *KIF11* + *KIF14* co-expression and any marker alone.

## 5. Conclusions

In conclusion, herein we provided the first evidence that KIF11 and KIF14 could be defined as positive prognostic markers in PDAC based on the protein-based IHC assay, which is a widely available and clinically used tool for prognostic and predictive testing. Furthermore, as tumor molecular portraits are also of clinical importance, our study reveals valuable data on the significant association of high *KIF11* and *KIF14* mRNA levels with a poor prognosis of PDAC patients, as well as on their putative, close functional relationship with one another, *CEP55*, *ASPM*, and *GAMT* genes. Specifically, stratification of PDACs with respect to combined expression profiles of these five molecular biomarkers (*KIF11*/*KIF14*/*CEP55*/*ASPM*/*GAMT*) may be clinically relevant, presenting a better performance than any marker alone. Our findings need to be validated in large-size, multicenter, and ideally prospective studies.

## Figures and Tables

**Table 1:** Characteristics of the study population by KIF11 immunoexpression groups.

Variables	*n* (%)	KIF11 IS		*p*-Value	KIF11 PS		*p*-Value	KIF11 IRS		*p*-Value
		↓*n* = 26 (38.24)	↑*n* = 42 (61.76)		↓*n* = 58 (85.29)	↑*n* = 10 (14.71)		↓*n* = 63 (92.65)	↑*n* = 5 (7.35)	
Age (years)										
≤ 60	29 (42.65)	12 (41.38)	17 (58.62)	0.80	22 (75.86)	7 (24.14)	0.09	25 (86.21)	4 (13.79)	0.16
> 60	39 (57.35)	14 (35.90)	25 (64.10)		36 (92.31)	3 (7.69)		38 (97.44)	1 (2.56)	
Gender										
Male	34 (50.00)	13 (38.24)	21 (61.76)	>0.99	28 (82.35)	6 (17.65)	0.73	31 (91.18)	3 (8.82)	>0.99
Female	34 (50.00)	13 (38.24)	21 (61.76)		30 (88.24)	4 (11.76)		32 (94.12)	2 (5.88)	
Grading										
G1	5 (7.35)	1 (20.00)	4 (80.00)	0.68	5 (100.0)	0 (0.00)	0.46	5 (100.00)	0 (0.00)	0.11
G2	55 (80.88)	22 (40.00)	33 (60.00)		47 (85.45)	8 (14.55)		52 (94.55)	3 (5.45)	
G3	8 (11.77)	3 (37.50)	5 (62.50)		6 (75.00)	2 (25.00)		6 (75.00)	2 (25.00)	
pT status										
T1	10 (15.87)	5 (50.00)	5 (50.00)	0.47	10 (100.0)	0 (0.00)	0.37	10 (100.0)	0 (0.00)	0.60
T2	43 (68.25)	13 (30.23)	30 (69.77)		37 (86.05)	6 (13.95)		39 (90.70)	4 (9.30)	
T3-T4	10 (15.87)	4 (40.00)	6 (60.00)		8 (80.00)	2 (20.00)		9 (90.00)	1 (10.00)	
pN status										
N0	30 (45.46)	9 (30.00)	21 (70.00)	0.21	25 (83.33)	5 (16.67)	0.72	27 (90.00)	3 (10.00)	0.32
N1-N2	36 (54.54)	17 (47.22)	19 (52.78)		32 (88.89)	4 (11.11)		35 (97.22)	1 (2.78)	
TNM stage										
I	24 (38.71)	6 (25.00)	18 (75.00)	0.20	20 (83.33)	4 (16.67)	0.29	22 (91.67)	2 (8.33)	0.54
II	24 (38.71)	12 (50.00)	12 (50.00)		21 (87.50)	3 (12.50)		22 (91.67)	2 (8.33)	
III-IV	14 (22.58)	5 (35.71)	9 (64.29)		14 (100.0)	0 (0.00)		14 (100.0)	0 (0.00)	
Location										
Head	60 (88.24)	23 (38.33)	37 (61.67)	>0.99	53 (88.33)	7 (11.67)	0.09	56 (93.33)	4 (6.67)	0.48
Body-tail	8 (11.76)	3 (37.50)	5 (62.50)		5 (62.50)	3 (37.50)		7 (87.50)	1 (12.50)	
VI										
Absent	39 (72.22)	17 (43.59)	22 (56.41)	>0.99	33 (84.62)	6 (15.38)	>0.99	37 (94.87)	2 (5.13)	>0.99
Present	15 (27.78)	7 (46.67)	8 (53.33)		13 (86.67)	2 (13.33)		14 (93.33)	1 (6.67)	
PNI Absent Present	22 (34.38)	10 (45.45)	12 (54.55)	0.59	16 (72.73)	6 (27.27)	0.05	19 (86.36)	3 (13.64)	0.11
	42 (65.62)	15 (35.71)	27 (64.29)		39 (92.86)	3 (7.14)		41 (97.62)	1 (2.38)	

Abbreviations: IS—immunointensity; PS—immunopercentage; IRS—immunoreactive score; VI—vascular invasion; PNI—perineural invasion. Chi-square or Fisher’s exact test.

**Table 2:** Characteristics of the study population by KIF14 immunoexpression groups.

Variables	*n* (%)	KIF14 IS		*p* -Value	KIF14 PS		*p*-Value	KIF14 IRS		*p*-Value
		↓ *n* = 21 (30.88)	↑ *n* = 47 (69.12)		↓ *n* = 31 (45.59)	↑ *n* = 37 (54.41)		↓ *n* = 30 (44.12)	↑ *n* = 38 (55.88)	
Age (years)										
≤ 60	29 (42.65)	8 (27.59)	21 (72.41)	0.79	14 (48.28)	15 (51.72)	0.81	14 (48.28)	15 (51.72)	0.63
> 60	39 (57.35)	13 (33.33)	26 (66.67)		17 (43.59)	22 (56.41)		16 (41.03)	23 (58.97)	
Gender										
Male	34 (50.00)	8 (23.53)	26 (76.47)	0.29	15 (44.12)	19 (55.88)	>0.99	13 (38.24)	21 (61.76)	0.46
Female	34 (50.00)	13 (38.24)	21 (61.76)		16 (47.06)	18 (52.94)		17 (50.00)	17 (50.00)	
Grading										
G1	5 (7.35)	1 (20.00)	4 (80.00)	0.10	3 (60.00)	2 (40.00)	0.40	2 (40.00)	3 (60.00)	0.15
G2	55 (80.88)	20 (36.36)	35 (63.64)		26 (47.27)	29 (52.73)		27 (49.09)	28 (50.91)	
G3	8 (11.77)	0 (0.00)	8 (100.0)		2 (25.00)	6 (75.00)		1 (12.50)	7 (87.50)	
pT status										
T1	10 (15.87)	4 (40.00)	6 (60.00)	0.56	6 (60.00)	4 (40.00)	0.40	5 (50.00)	5 (50.00)	0.83
T2	43 (68.25)	11 (25.58)	32 (74.42)		19 (44.19)	24 (55.81)		17 (39.54)	26 (60.47)	
T3-T4	10 (15.87)	2 (20.00)	8 (80.00)		3 (30.00)	7 (70.00)		4 (40.00)	6 (60.00)	
pN status										
N0	30 (45.46)	8 (26.67)	22 (73.33)	0.44	18 (60.00)	12 (40.00)	0.08	13 (43.33)	17 (56.67)	0.81
N1-N2	36 (54.54)	13 (36.11)	23 (63.89)		13 (36.11)	23 (63.89)		17 (47.22)	19 (52.78)	
TNM stage										
I	24 (38.71)	6 (25.00)	18 (75.00)	0.43	15 (62.50)	9 (37.50)	0.12	10 (41.67)	14 (58.33)	0.86
II	24 (38.71)	6 (25.00)	18 (75.00)		8 (33.33)	16 (66.67)		10 (41.67)	14 (58.33)	
III-IV	14 (22.58)	6 (42.86)	8 (57.14)		6 (42.86)	8 (57.14)		7 (50.00)	7 (50.00)	
Location										
Head	60 (88.24)	19 (31.67)	41 (68.33)	>0.99	28 (46.67)	32 (53.33)	0.72	26 (43.33)	34 (56.67)	0.72
Body-tail	8 (11.76)	2 (25.00)	6 (75.00)		3 (37.50)	5 (62.50)		4 (50.00)	4 (50.00)	
VI										
Absent	39 (72.22)	14 (35.90)	25 (64.10)	0.18	20 (51.28)	19 (48.72)	>0.99	19 (48.72)	20 (51.28)	0.37
Present	15 (27.78)	2 (13.33)	13 (86.67)		7 (46.67)	8 (53.33)		5 (33.33)	10 (66.67)	
PNI Absent Present	22 (34.38)	5 (22.73)	17 (77.27)	0.57	10 (45.45)	12 (54.55)	>0.99	9 (40.91)	13 (59.09)	>0.99
	42 (65.62)	14 (33.33)	28 (66.67)		19 (45.24)	23 (54.76)		18 (42.86)	24 (57.14)	

Abbreviations: IS—immunointensity; PS—immunopercentage; IRS—immunoreactive score; VI—vascular invasion; PNI—perineural invasion. Chi-square or Fisher’s exact test.

**Table 3:** Univariate Cox proportional hazards models for overall survival of pancreatic ductal adenocarcinoma patients.

Variable	Univariate Analysis			
	HR	95% CI		*p*-Value
		Lower	Upper	
KIF11 IRS (low vs. high)	0.23	0.06	0.96	**0.04**
KIF11 IS (low vs. high)	1.49	0.82	2.71	0.19
KIF11 PS (low vs. high)	0.41	0.17	0.98	**0.045**
KIF14 IRS (low vs. high)	0.44	0.24	0.79	**0.006**
KIF14 IS (low vs. high)	0.58	0.32	1.06	0.08
KIF14 PS (low vs. high)	0.70	0.39	1.26	0.24
Age (≤60 vs. ≥60)	1.14	0.63	2.07	0.67
Gender (female vs. male)	0.97	0.54	1.75	0.93
Grade (G1 vs. G2-G3)	2.12	0.51	8.00	0.30
pN (absent vs. present)	1.27	0.70	2.32	0.44
pT (T1-T2 vs. T3-T4)	1.08	0.48	2.45	0.85
Stage (I-II vs. III-IV)	2.05	1.02	4.13	**0.04**
PNI (absent vs. present)	1.47	0.79	2.76	0.23
VI (absent vs. present)	2.41	1.17	4.98	**0.02**

Abbreviations: HR, hazard ratio; CI, confidence interval; IS—immunointensity; PS—immunopercentage; IRS—immunoreactive score; VI—vascular invasion; PNI—perineural invasion. Bold values denote statistically significant *p*-values (*p* < 0.05).

**Table 4:** Multivariate Cox proportional hazards models for overall survival of pancreatic ductal adenocarcinoma patients.

Variable	Multivariate Analysis: IRS				Multivariate Analysis: IS				Multivariate Analysis: PS			
	HR	95% CI		*p*	HR	95% CI		*p*	HR	95% CI		*p*
		Lower	Upper			Lower	Upper			Lower	Upper	
KIF11 IRS (low vs. high)	0.06	0.005	0.64	**0.02**	-	-	-	-	-	-	-	-
KIF11 IS (low vs. high)	-	-	-	-	3.05	1.29	7.19	**0.01**	-	-	-	-
KIF11 PS (low vs. high)	-	-	-	-	-	-	-	-	0.31	0.06	1.53	0.15
KIF14 IRS (low vs. high)	0.20	0.08	0.51	**0.001**	-	-	-	-	-	-	-	-
KIF14 IS (low vs. high)	-	-	-	-	0.25	0.10	0.67	**0.006**	-	-	-	-
KIF14 PS (low vs. high)	-	-	-	-	-	-	-	-	0.45	0.17	1.21	0.11
Age (≤ 60 vs. ≥ 60)	1.15	0.50	2.64	0.74	1.61	0.69	3.73	0.27	1.71	0.68	4.25	0.25
Gender (female vs. male)	0.93	0.37	2.30	0.87	0.80	0.32	1.98	0.63	0.87	0.33	2.29	0.78
Grade (G1 vs. G2-G3)	3.00	0.24	37.98	0.40	6.00	0.47	76.79	0.17	4.64	0.44	48.57	0.20
pN (absent vs. present)	1.05	0.38	2.90	0.93	1.76	0.55	5.69	0.34	1.20	0.38	3.73	0.76
pT (T1-T2 vs. T3-T4)	2.17	0.66	7.14	0.20	2.06	0.68	6.21	0.20	1.91	0.63	5.73	0.25
Stage (I-II vs. III-IV)	2.06	0.73	5.77	0.17	1.43	0.48	4.24	0.52	1.91	0.68	5.31	0.22
PNI (absent vs. present)	0.74	0.29	1.91	0.54	1.27	0.48	3.36	0.64	0.74	0.27	2.03	0.56
VI (absent vs. present)	3.43	1.30	9.06	**0.01**	4.09	1.49	11.20	**0.006**	1.96	0.70	5.51	0.20

Abbreviations: HR, hazard ratio; CI, confidence interval; IS—immunointensity; PS—immunopercentage; IRS—immunoreactive score; VI—vascular invasion; PNI—perineural invasion. HR: adjusting for age, gender, histological grade, tumor stage, pT, pN, and study. “-“ indicates that the variable was not included in multivariate analysis. Bold values denote statistically significant *p*-values (*p* < 0.05).

**Table 5:** Characteristics of the TCGA cohort by *KIF11* and *KIF14* expression groups.

Variables	*n* (%)	*KIF11*		*p* -Value	*KIF14*		*p*-Value
		Negative *n* = 86	Positive *n* = 91		Negative *n* = 128	Positive *n* = 49	
Gender							
Male	97 (54.80)	45 (46.39)	52 (53.61)	0.55	69 (71.13)	28 (28.87)	0.74
Female	80 (45.20)	41 (51.25)	39 (48.75)		59 (73.75)	21 (26.25)	
Age							
≤60	59 (33.33)	31 (52.54)	28 (47.46)	0.52	42 (71.19)	17 (28.81)	0.86
>60	118 (66.67)	55 (46.61)	63 (53.39)		86 (72.88)	32 (27.12)	
Grading							
G1	31 (17.71)	22 (70.97)	9 (29.03)	**0.01**	30 (96.77)	1 (3.23)	**0.002**
G2	94 (53.71)	44 (46.81)	50 (53.19)		66 (70.21)	28 (29.79)	
G3-G4	50 (28.57)	19 (38.00)	31 (62.00)		30 (60.00)	20 (40.00)	
pT status							
T1-T2	30 (17.14)	18 (60.00)	12 (40.00)	0.17	24 (80.00)	6 (20.00)	0.37
T3-T4	145 (82.86)	66 (45.52)	79 (54.48)		102 (70.34)	43 (29.66)	
pN status							
N0	49 (28.49)	24 (48.98)	25 (51.02)	>0.99	38 (77.55)	11 (22.45)	0.35
N1	123 (71.51)	59 (47.97)	64 (52.03)		86 (69.92)	37 (30.08)	
TNM stage							
I	21 (12.00)	13 (61.90)	8 (38.10)	0.24	18 (85.71)	3 (14.29)	0.20
II-IV	154 (88.00)	71 (46.10)	83 (53.90)		108 (70.13)	46 (29.87)	

Significant *p*-values (*p* < 0.05) are marked in bold. Abbreviation: TCGA, the Cancer Genome Atlas.

**Table 6:** Univariate and multivariate Cox proportional hazards models for overall survival of TCGA patients with pancreatic adenocarcinoma.

Variable	Univariate Analysis				Multivariate Analysis: KIF11				Multivariate Analysis: KIF14			
	HR	95% CI		*p*	HR	95% CI		*p*	HR	95% CI		*p*
		Lower	Upper			Lower	Upper			Lower	Upper	
*KIF11* (low vs. high)	2.25	1.47	3.45	**0.0002**	1.75	1.13	2.70	**0.01**	-	-	-	-
*KIF14* (low vs. high)	2.99	1.97	4.54	**<0.0001**	-	-	-	-	2.65	1.70	4.15	**<0.0001**
Age (≤ 60 vs. ≥ 60)	1.41	0.90	2.21	0.13	1.24	0.77	1.98	0.38	1.28	0.80	2.04	0.31
Gender (female vs. male)	0.81	0.54	1.23	0.33	0.78	0.51	1.20	0.26	0.84	0.54	1.30	0.41
Grade (G1 vs. G2-G3)	2.18	1.15	4.13	**0.02**	1.53	0.80	2.95	0.20	1.21	0.61	2.39	0.59
pN (absent vs. present)	2.10	1.25	3.52	**0.005**	2.00	1.15	3.47	**0.01**	2.09	1.19	3.67	**0.01**
pT (T1-T2 vs. T3-T4)	2.21	1.14	4.28	**0.02**	1.34	0.65	2.75	0.43	1.29	0.63	2.65	0.49
Stage (I-II vs. III-IV)	0.74	0.23	2.34	0.60	-	-	-	-	-	-	-	-

Significant *p*-values (*p* < 0.05) are marked in bold. Abbreviations: CI, confidence interval; HR, hazard ratio; TCGA, the Cancer Genome Atlas. HR: adjusting for age, gender, histological grade, pN, pT, and each marker separately. Owing to the strong correlations between KIF11 and KIF14 mRNA levels, they were not included in the same multivariate model. “-“ indicates variable was not included in multivariate Cox analysis.

## Data Availability

Publicly available datasets were analyzed in this study. These data can be found here: http://www.cbioportal.org/study/summary?id=paad_tcga_pan_can_atlas_2018 (accessed on 26 August 2020); https://xenabrowser.net (accessed on 26 August 2020); http://ualcan.path.uab.edu/ (accessed on 19 April 2021). Our own data presented in this study are available on request from the corresponding author. The data are not publicly available due to ethical restrictions.

## Supplementary Materials

The following are available online at https://www.mdpi.com/article/10.3390/cancers13123017/s1: Figure S1: Heatmaps of Spearman correlation analysis. Figure S2: Kaplan–Meier curves for overall survival of pancreatic ductal adenocarcinoma patients stratified by (A) combined KIF11 and KIF14 immunoexpression. The KIF11 + KIF14 score was formed based on a total immunoexpression score (IRS), as described in Section 2; (B) KIF11 staining intensity (IS) score as continuous variable; (C) KIF11 percentage (PS) score as continuous variable. (D) KIF11 staining intensity (IS) scores with cut points at  ≥ 2.5. *p*-values were calculated using the log-rank test. Figure S3: Kaplan–Meier curves for overall survival of pancreatic adenocarcinoma patients of the TCGA cohort stratified by (A) KIF11 and KIF14 co-expression (red line: KIF11^high^/KIF14^high^ vs. blue line: KIF11^low^/KIF11^low^); (B) KIF11, KIF14, CEP55, ASPM and GAMT co-expression (red line: KIF11^high^/KIF14^high^/CEP55^high^/ASPM^high^/GAMT^low^ vs. blue line: KIF11^low^/KIF14^low^/CEP55^low^/ASPM^low^/GAMT^high^). *p*-Values were calculated using the log-rank test. Table S1: Descriptive statistics for KIF11 and KIF14 expression parameters. Table S2: Univariate and multivariate Cox proportional hazards models for overall survival of TCGA patients with pancreatic adenocarcinoma.
