# Supplementary material for: Prognostic Significance of KIF11 and KIF14 Expression in Pancreatic Adenocarcinoma
Source: Cancers (Basel). 2021 Jun 16;13(12):3017. doi: 10.3390/cancers13123017 (PMC8234517; doi:10.3390/cancers13123017)
Supplement: Supplementary file 1 [file cancers-13-03017-s001.zip › supplementary table.pdf]

Table S1. Descriptive statistics for KIF11 and KIF14 expression parameters.

	TUMOR						ADJACENT					
Variable	MED	IQR	MIN	MAX	Mean	SD	MED	IQR	MIN	MAX	Mean	SD
KIF11 IS	2	1-2	0	3	1.58	0.86	1	0-1	0	2	0.59	0.53
KIF11 PS	1	0-1.46	0	3	0.88	0.82	3	0-4	0	4	2.03	1.85
KIF11 IRS	1	0-2.23	0	9	1.54	1.82	3	0-4	0	4	2.06	1.87
KIF14 IS	1.5	1-2	0	3	1.70	0.70	1	1-1	0	2	0.98	0.40
KIF14 PS	4	3-4	0	4	3.40	0.85	4	4-4	0	4	3.42	1.26
KIF14 IRS	6	4-8	0	12	6.03	2.90	4	4-4	0	8	3.68	1.64

IS – immunointensity; PS - immunopercentage; IRS – immunoreactive score (IS x PS); med – median; IQR-interquartile range; min- minimum; max – maximum; SD – standard deviation.

Table S2. Univariate and multivariate Cox proportional hazards models for overall survival of TCGA patients with pancreatic adenocarcinoma.

Variable	Univariate analysis				Multivariate analysis: KIF11+KIF14*				Multivariate analysis: 5-gene panel**			
	HR	95% CI		*P*	HR	95% CI		*P*	HR	95% CI		*P*
		lower	upper			lower	upper			lower	upper	
*KIF11+KIF14**	3.41	2.12	5.47	**<0.0001**	2.60	1.58	4.29	**0.0002**	-	-	-	-
5-gene panel**	5.18	2.63	10.20	**<0.0001**	-	-	-	-	5.21	2.41	11.27	**<0.0001**
ASPM (low vs. high)	2.61	1.73	3.94	**<0.0001**	-	-	-	-	-	-	-	-
CEP55 (low vs. high)	2.49	1.58	3.90	**<0.0001**	-	-	-	-	-	-	-	-
GAMT (high vs. low)	0.61	0.39	0.94	**0.03**	-	-	-	-	-	-	-	-
Age (≤ 60 vs. ≥ 60)	1.41	0.90	2.21	0.13	1.46	0.85	2.50	0.17	2.68	1.20	5.97	**0.02**
Sex (female vs. male)	0.81	0.54	1.23	0.33	0.92	0.56	1.49	0.72	1.00	0.50	2.05	0.99
Grade (G1 vs. G2-G3)	2.18	1.15	4.13	**0.02**	1.68	0.67	4.19	0.27	1.20	0.39	3.67	0.75
pN (absent vs. present)	2.10	1.25	3.52	**0.005**	2.45	1.28	4.69	**0.01**	4.37	1.49	12.80	**0.01**
pT (T1-T2 vs. T3-T4)	2.21	1.14	4.28	**0.02**	1.08	0.50	2.36	0.84	1.79	0.39	8.14	0.45

Significant p-values ($p < 0.05$) are marked in bold.
*n = 133; $KIF11^{low}/KIF14^{low}$ vs. $KIF11^{high}/KIF14^{high}$
**n = 85; $KIF11^{low}/KIF14^{low}/CEP55^{low}/ASPM^{low}/GAMT^{high}$ *vs.* $KIF11^{high}/KIF14^{high}/CEP55^{high}/ASPM^{high}/GAMT^{low}$)
Abbreviations: CI, confidence interval; HR, hazard ratio; OS – overall survival; TCGA – The Cancer Genome Atlas. HR: adjusting for age, gender, histologic grade, pN, pT, and each marker separately. Owing to the strong correlations between *KIF11* and *KIF14* mRNA expression, they were not included in the same multivariate model.
'-' indicates variable was not included in multivariate Cox analysis.
