# Supplementary figures and images for: Prognostic Significance of KIF11 and KIF14 Expression in Pancreatic Adenocarcinoma
Source: Cancers (Basel). 2021 Jun 16;13(12):3017. doi: 10.3390/cancers13123017 (PMC8234517; doi:10.3390/cancers13123017)

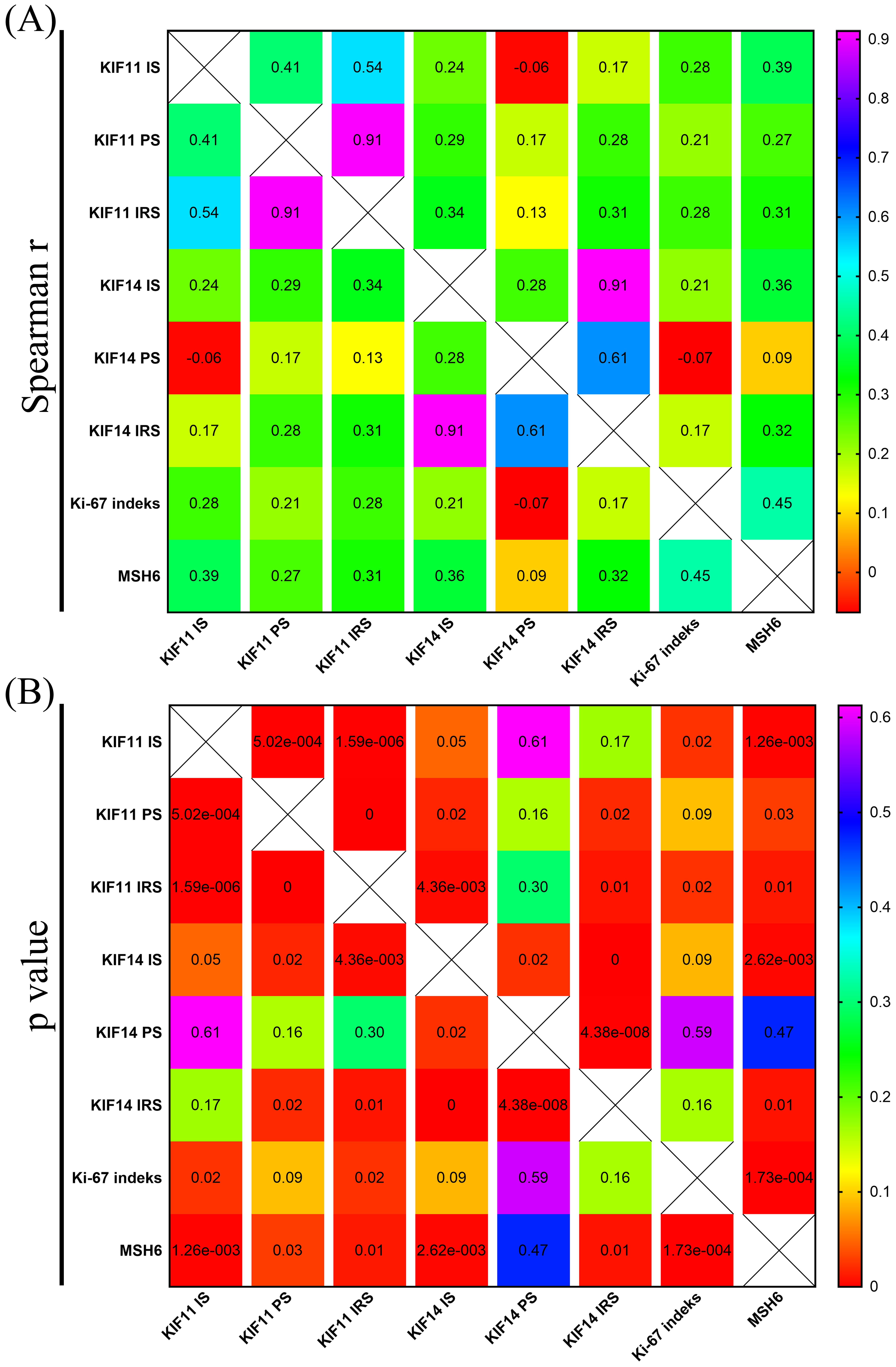

Supplement: Supplementary file 1 [file cancers-13-03017-s001.zip › Figure S1.tif]

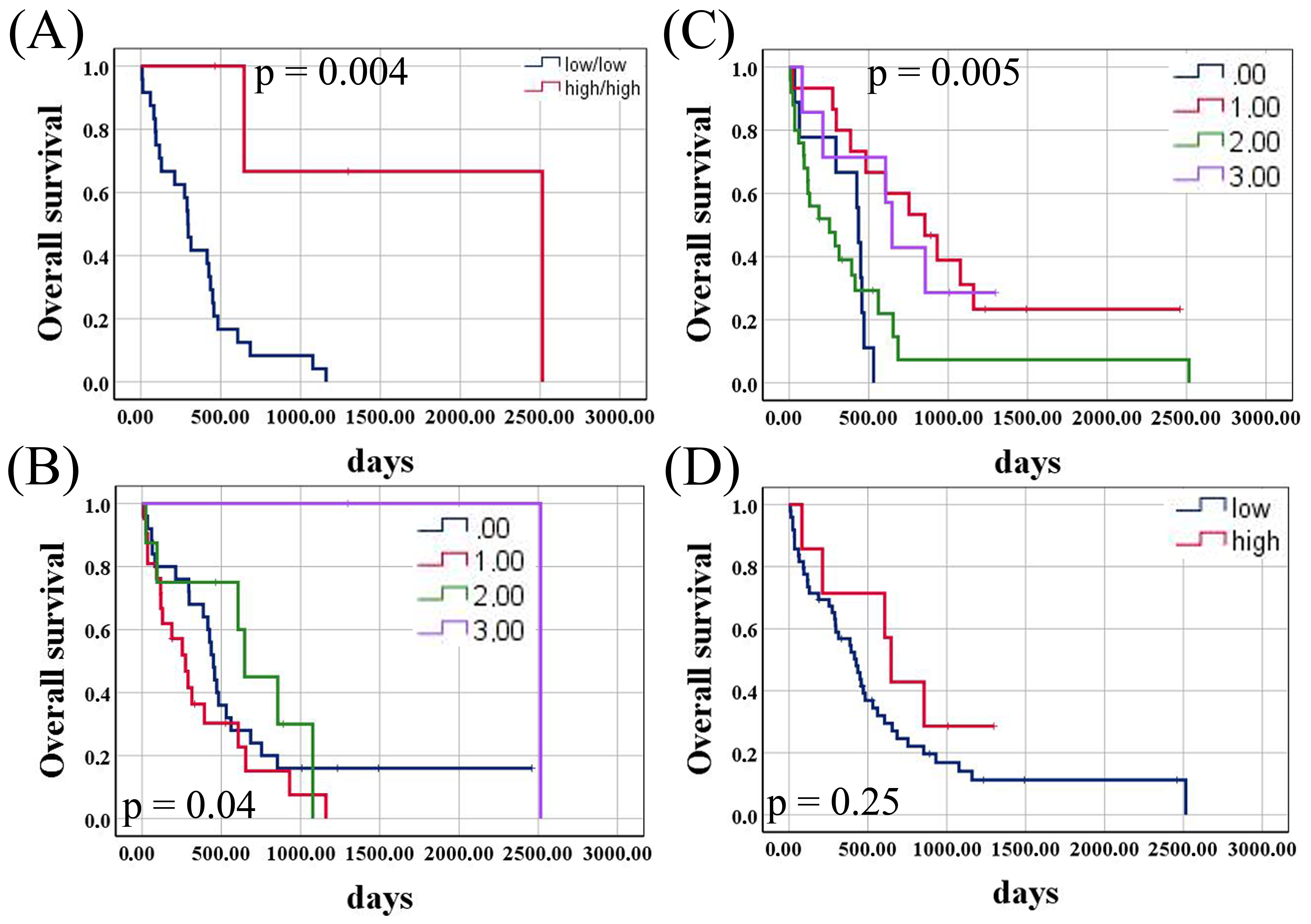

Supplement: Supplementary file 1 [file cancers-13-03017-s001.zip › Figure S2.tif]

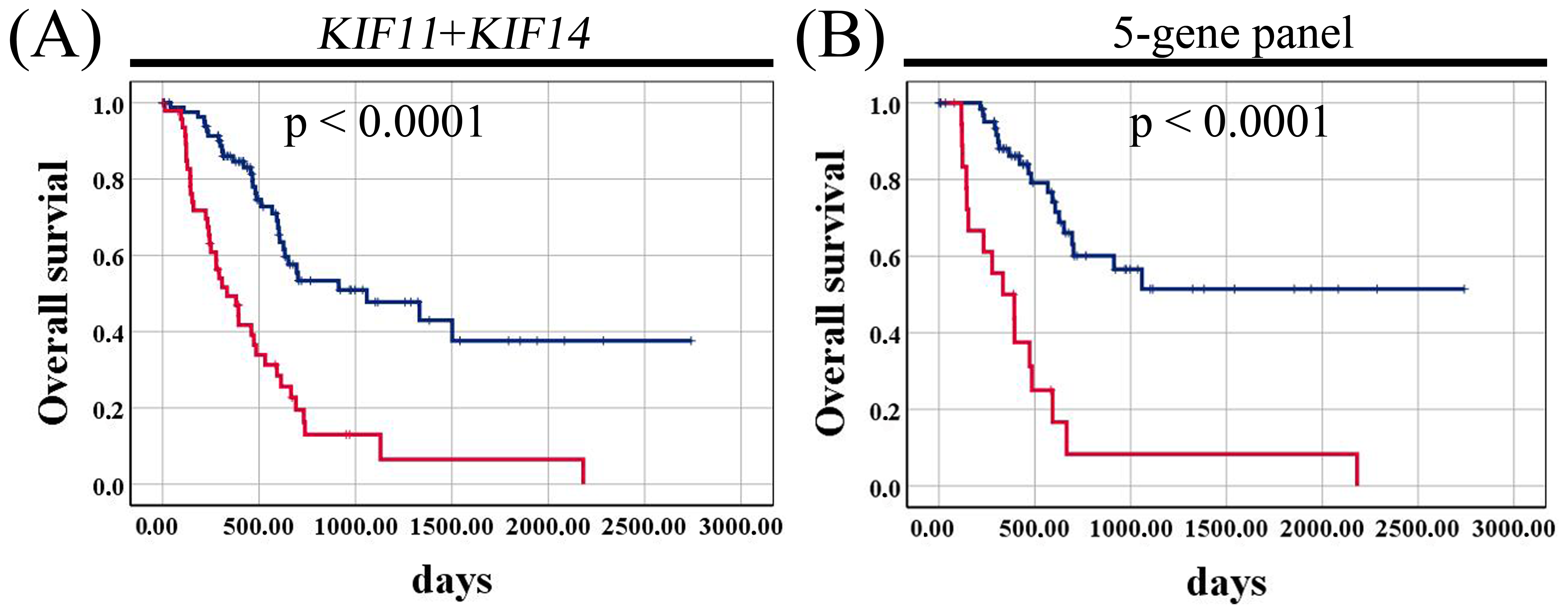

Supplement: Supplementary file 1 [file cancers-13-03017-s001.zip › Figure S3.tif]
